# Cell-free supernatant of *Devosia* sp. (strain SL43) mitigates the adverse effects of salt stress on soybean (*Glycine max* L.) seed vigor index

**DOI:** 10.3389/fpls.2023.1071346

**Published:** 2023-03-28

**Authors:** Nadia Monjezi, Iraj Yaghoubian, Donald L. Smith

**Affiliations:** Department of Plant Science, McGill University, St Anne-de-Bellevue, QC, Canada

**Keywords:** soybean, salinity, *Devosia* sp., cell-free supernatants, seed germination, vigor index, culture medium optimization

## Abstract

Soil salinity is a major constraint for soybean production worldwide, and the exploitation of plant growth-promoting bacteria (PGPB) and their bioactive metabolite(s) can improve plant salinity tolerance. With this objective, two experiments were performed, aiming to test 4 culture media (YEM(A), TYE(A), TS(A), and LB(A)) for growing a novel *Devosia* sp. (strain SL43), and then evaluating cell-free supernatants (CFS) from the *Devosia* sp. on germination of soybean (*Glycine max* L.) seeds under salinity stress. Soybean seeds were subjected to three salinity levels (0, 100, and 125 mM NaCl) and 6 levels of *Devosia* sp. CFS dilution (0, 1:1, 1:100, 1:250, 1:500, 1:1000). The results indicated that 125 mM NaCl concentration caused the greatest reduction in the total number of germinated seeds (15%), germination rate (43.6%), root length (55.2%), root weight (39.3%), and seed vigor (68%), and it also increased mean germination time by 71.9%. However, *Devosia*-CFS improved soybean germination, and the greatest effect was obtained at 1:1 dilution. Under the highest salinity level, application of CFS at 1:1 dilution increased final germination (17.6%), germination rate (18.6%), root length (162.2%), root weight (239.4%), seed vigor index (318.7%), and also shortening mean germination time by 19.2%. The results indicated that seed vigor index was positively correlated with other traits except for mean germination time. Our study suggested that the highest productivity of Devoisa sp. was obtained from the YEM medium. Results also suggested that CFS produced by the novel Devosia sp. (SL43 strain) can successfully alleviate salt stress effects on soybean seed germination and manipulating the chemical composition of the growth medium can influence the effectiveness of these bioactive metabolites.

## 1 Introduction

Soybean (*Glycine max* L.) is one of the world’s more widely produced crops, due to its high nutritional value, which plays a vital role in global food security (Waqas et al., 2014; Chung et al., 2020). Previous studies have shown that soybean growth, production and quality are strongly influenced by abiotic stresses (Chung et al., 2020; Yaghoubian et al., 2021).

As our climate continues to be affected by global warming/climate change, the consequences of that warming grow more intense, and the frequency of extreme weather events increases. This shifting of weather patterns is the biggest challenge currently facing farmers and farm communities worldwide (Kheiri et al., 2021; Clarke et al., 2022). Rising demand for agricultural commodities persists despite continuous warming, which is driving the agricultural sector seek new ways of crop production (Chandio et al., 2020; Yadav et al., 2021). Further, climate-induced environmental stressors such as salinity, drought, and heat are among the principal factors reducing crop productivity worldwide, which diminishes global food security and environmental sustainability. The situation with regard to soil salinization is worsening substantially faster than researchers had predicted less than a decade ago, putting the world on alert for the potential spread of salinity issues into currently unaffected regions (Mukhopadhyay et al., 2021). Salinity impairs many plant functions, from the seed germination stage to final seed production; seedling emergence at the initiation phase of the lifecycle of plants is highly susceptible to salinity (Naamala et al., 2022; Yaghoubian et al., 2022a). However, once the plants are overcome the seedling stage, they are better able to cope with the adverse consequences of salinity stress (Gholizadeh et al., 2021; Shah et al., 2022).

Building climate resilience requires relying on sustainable farming methods and practices in the face of climate change; hopefully these technologies can relieve pressure on the environment, cut greenhouse gas emissions, and also aid in managing future risks (Dhankher and Foyer, 2018; Adegbeye et al., 2020; Yadav et al., 2021). One of the interesting methods for increasing the resilience of our food systems is linking farming communities and scientists together to work toward easing dependence on chemical products while increasing reliance on eco-friendly product options such as plant growth-promoting bacteria (PGPB), which are increasingly being employed as bio-stimulant formulations, promoting plant health, development, and sustainability (Naamala and Smith, 2020; Fiodor et al., 2021; Yaghoubian et al., 2022b). There are various mechanisms by which microbes effectively promote plant growth in saline soil. Several studies have shown that beneficial microbes can directly improve plant growth under salinity stress, perhaps by synthesizing specific growth-stimulating hormones such as auxins, cytokinin, and gibberellins or by downregulation ethylene formation. Indeed, this kind of hormonal coordination by PGPB will result in minimizing plant ethylene levels and therefore reducing the detrimental and inhibitory effects of this phytohormone on plant growth under salt stress. On the other hand, some beneficial microbes might either promote plant growth through bacterial auxin production or increase the production of endogenous plant auxins, which will result in controlling primary root elongation and boost lateral root formation, and finally, the equilibrium between ethylene and auxin enables plants to uptake water, ions, and nutrients more efficiently under salinity stress (Iqbal et al., 2017; Kudoyarova et al., 2019; Kumar et al., 2020; Eichmann et al., 2021; Park et al., 2021). Therefore, optimizing germination such that it can largely overcome soil salinity effects is possible by utilizing PGPB, a promising tool for a more sustainable future. However, the crop colonization ability of most beneficial bacteria varies depending upon the host, bacterial species, and salt concentration (Miljaković et al., 2022; Nigam et al., 2022; Sulastri et al., 2022; Yaghoubian et al., 2022a). Moreover, bacterial trait–environment relationships can vary from farm to farm and from region to region, which may affect the efficacy of PGPB (Goddard et al., 2001; Meena et al., 2015). The most recent approach, within market limitations, for PGPB strain technologies, is using Cell-Free Supernatants (CFSs) of beneficial PGPB, which could offer innovative alternatives in dealing with these challenging limitations, as well as promoting crop productivity (Pellegrini et al., 2020; Naamala and Smith, 2021). Such microbial-derived mixtures may include growth hormones, secondary metabolites, various signal compounds, and antioxidant enzymes, which would positively enhance plant growth (Yasmin et al., 2004; Fiodor et al., 2021; Saberi Riseh et al., 2021). Thus, CFS technologies offer the most viable hope for enhancing crop production under a challenging climate change situation, due to a range of conditions and the probability of success of microbial compounds which are less affected by variable environmental conditions (Naamala and Smith, 2020; Shah et al., 2022). Although these CFS metabolites have recently gained greater attention, a meaningful gap remains in the progression from research to implementation in existing farming systems. The most critical step is determining the type of growth medium for *in vitro* cultivation because it consistently influences microbial organic compound production. While plenty of studies primarily focus on the growth and nutritional requirements of root nodule bacteria, less attention has been focused on maximizing growth rates and increasing production of plant growth-promoting compounds. An appropriate medium contains a good source of carbon, nitrogen, mineral salts, and growth factors; nutrient media such as yeast extract mannitol (YEM) and tryptone yeast extract (TYE) medium are found to be very suitable for growing PGPB. Indeed, although there are many enrichment culture formulations, each bacterium has entirely unique nutritional requirements, and testing culture medium and new culture conditions can make it possible to find novel bacterial compounds enhancing plant growth and development (Pastor-Bueis et al., 2017; Sessitsch et al., 2019; Trianto et al., 2020; Jiao et al., 2021; Yusfi et al., 2021).

Thus, we hypothesized that growth conditions can play a vital role in supporting plant growth-promoting compound production. Therefore, the present study focused on acquiring detailed knowledge and understanding of cost-effective technologies to produce such biostimulants and reduce detrimental impacts of salinity on soybean germination.

## 2 Materials and methods

### 2.1 Experimental design

This research was conducted in three sets of experiments at the Macdonald Campus of McGill University in 2022. The first study examined bacterial growth on a set of agar culture media; the best medium was selected based on growth rate. Then, the growth of bacteria in the suspension culture of the selected medium was monitored using Cytation instrumentation (Cytation 5^th^ Cell Imaging Multimode Reader, BioTek Instruments, Inc.). The second and third experiments examined simultaneously the effectiveness of Cell-free supernatant (CFS) harvested from bacterial suspensions of Yeast Extract Mannitol Broth (YEMB) and Tryptone Yeast Extract Broth (TYEB) media as technologies for enhancing seed germination of soybean (*Glycine max* L. var P09A62X) under saline and non-saline conditions. The treatments were determined as factorial combinations of three NaCl levels (0, 100, and125 mM) and dilution ratios of 1:100, 1:250, 1:500, and 1:1000 for the CFS (1 mL *Devosia* sp. CFS and 1, 100, 250, 500, and 1000 mL distilled water, respectively). Each experiment was organized following a completely randomized design.

### 2.2 Evaluation of solid and broth culture medium

The genus *Devosia* is a member of the *Alphaproteobacteria* and is a motile and gram-negative bacterium classified within the family *Hyphomicrobiaceae* of the order Rhizobiales (Talwar et al., 2020). *Devosia* sp. strain SL43 isolated from root nodules of *Amphicarpaea bracteate* is a plant growth-promoting phytomicrobiome member which was isolated from an undomesticated legume native to southwestern Quebec, through previous research in our laboratory (Ilangumaran et al., 2021); stock cultures were maintained on YEM broth slants at -80°C and subcultured every three months. A total of 4 different culture medium compositions, including YEM(A) (Yeast extract - Mannitol Agar), TYE(A) (Tryptone Yeast Extract Agar), TS(A) (Tryptic Soy Agar), and LB(A) (Luria-Bertani Agar) were used for growing the bacterium. A loopful (0.1 mL) of a suspension of *Devosia* sp. strain SL43 was streaked onto four plates containing YEM(A), TYE(A), TS(A), and LB(A) inside a laminar air flow hood and incubated at 28 ± 2°C and the visual culture characteristics of SL43 colonies on the plates was observed once daily for 10 days; each experiment was performed three times. Growth was not observed on TS(A) and LB(A) media; however, significant bacterial growth was observed for YEM(A) and TYE(A) plates which were selected for the next steps. Pure cultures of SL43 on YEM(A) and TYE(A) plates were picked off the plates with a sterile inoculating loop and inoculated into 25 mL of YEM(B) or TYE(B), which were agitated on a rotary shaker at 150 rpm and 28°C until reaching maximum growth based on the optical density of the bacterial growth which was measured spectrophotometrically at 600 nm (Ultrospec 4300 pro UV/Visible Spectrophotometer, Biochrom, Ltd, Cambridge, UK).

Bacterial growth was monitored using a microplate reader (Cytation 5 Cell Imaging Multi-Mode Reader) by incubating *Devosia* sp. strain SL43 in a set of sterilized 96-well clear bottom microplates (Corning Incorporated, NY). Each row contained the following treatments, in randomized order with 12 replications: YEM(B) (Control), TYE(B) (Control), YEM(B) (distilled water), YEM(B) (100 mM NaCl), YEM(B) (125 mM NaCl), TYE(B) (distilled water), TYE(B) (100 mM NaCl), TYE(B) (125 mM NaCl).

Briefly, for measuring bacterial growth, 200 μL of the prepared bacterial cultures in YEM or TYE broth culture (prepared with distilled water or saline solution) were injected into the microplate wells. Additionally, two rows were allocated to the YEM(B) or TYE(B) without adding any bacterial starter cultures. After covering microplates with pre-processed lids, they were placed on a rotary shaker microplate reader at 355 rpm at 28°C. The optical density (OD) of the bacterium was monitored at 600 nm. The OD of each well was read every 2 h for 7 days.

### 2.3 Propagation of bacteria and harvesting cell-free supernatants

For germination tests, 100 µL of SL43 broth from the final step were inoculated into a 100 mL Erlenmeyer containing 50 mL of YEM or TYE broth medium for 10 days at 28 ± 2°C; the material was considered ready for germination evaluation when the O.D. reached 1.0 (or maximum growth); at this stage, cell-free supernatant (CFS) was collected by centrifuging the liquid culture at 10,000 g for 30 min at room temperature, in order to remove the cells and other larger particles; after being centrifuged, the supernatant was filtered through a 0.22-um pore size syringe membrane (AwelTM MF 48-R, NuAire, USA) (Legesse, 2016; Sarbadhikary and Mandal, 2017).

### 2.4 Seed germination test

Soybean seeds were prepared through surface sterilization in 5% NaOCl (sodium hypochlorite) for one minute, followed by rinsing three times in sterile distilled water to disinfect soybean seeds; then seeds were placed on filter paper in Petri dishes. After which surface-sterilized seeds were moistened with 5 mL of cell-free supernatant of strain SL43 at various dilution levels (control, 1:100, 1:500, and 1:1000), diluted in sterile distilled water (unstressed) or 100 and 125 mM NaCl solution (stressed). Additionally, in some Petri dishes, seeds were wetted with 5 mL of sterile distilled water (unstressed) or 100 and 125 mM NaCl solution (stressed) without CFS addition, acting as the control. Then, Petri dishes were put in a thin polyethylene bag to avoid drying caused by evaporation, and Petri dishes containing the treated seeds were placed inside a growth chamber and incubated at 25 ± 2°C with a relative humidity of 70% in darkness. The seed germination rate was observed several times per day over the following 96 h; a seed was considered germinated when the root was over 0.2 cm long. The number of germinated seeds and germination time of each seed was determined (Ghassemi-Golezani et al., 2016). In addition, root length and dry weight of seedlings were recorded 7 days after sowing. Other variables calculated are described immediately below.

### 2.5 Mean germination time

Computation of mean germination time (MGT) was performed according to the following formula:


$$MGT=\frac{\sum \left(D\times g\right)}{\sum n}$$

Where g is the number of seeds germinated on each day, D is the number of days from the start of the germination test, and n is the total number of seeds germinated at the termination of the experiment (Ellis and Roberts, 1981).

### 2.6 Germination rate

The germination rate (GR) was calculated followed Al-Mudaris (1998) as:


$$GR=\frac{\sum n}{\sum \left(D\times g\right)}$$

### 2.7 Seed vigor index

The seed vigor index (SVI) was computed using the formulas proposed by Yaghoubian et al. (2022c):


$$SVI=\frac{SDW}{MGT}$$

Where MGT is mean germination time and SDW is the mean value of seedling dry weight.

### 2.8 Statistical analysis

Data were analyzed using SAS 9.4, and differences between control and treatments were considered statistically significant at P < 0.05 using a Duncan’s multiple test. Excel software was used to draw figures. The correlations were calculated by the correlation (CORR) procedure of the SAS 9.4, software.

## 3 Results

### 3.1 Performance of solid and broth culture media

**Figure 1:**
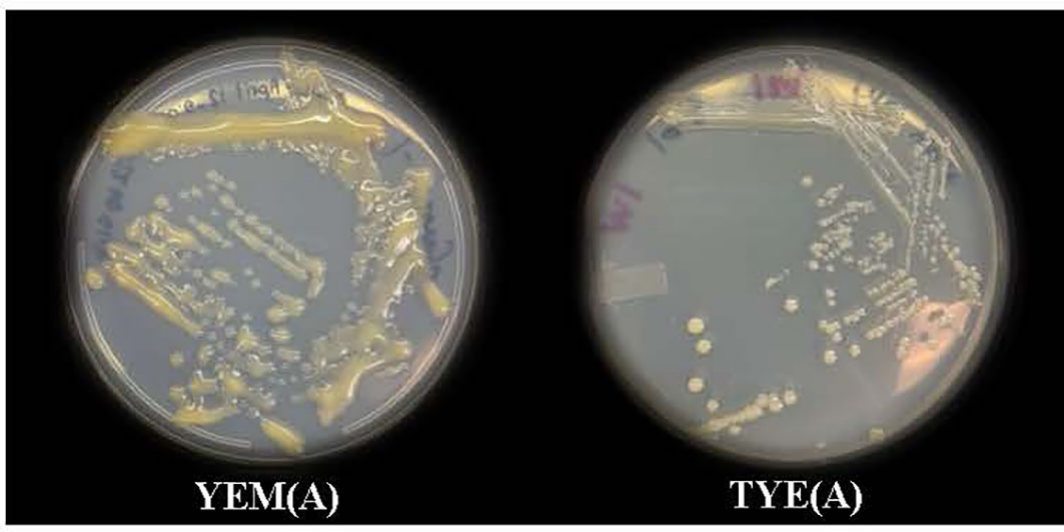
Growth of Devosia sp. (SL43 strain) in YEM(A) (Yeast Extract Mannitol Agar) and TYE(A) (Tryptone Yeast Extract Agar) media.

**Figure 2:**
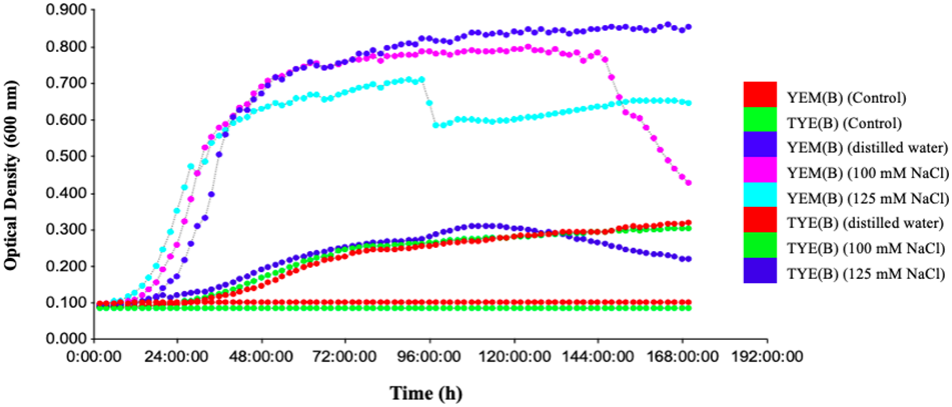
Changes in growth of *Devosia* sp. (SL43 strain) in Yeast Extract Mannitol broth (YEM-B) and Tryptone Yeast Extract broth (TYE-B) under salinity conditions. 1: YEM(B) (Control), 2: TYE(B) (Control), 3: YEM(B) (distilled water), 4: YEM(B) (100 mM NaCl), 5: YEM(B) (125 mM NaCl), 6: TYE(B) (distilled water), 7: TYE(B) (100 mM NaCl) and 8: TYE(B) (125 mM NaCl).

*Devosia* sp. on TS and LB Agar (A) media showed no visible growth. However, it grew well on YEM and TYE (A) media at pH 7.0 as seen after incubation for 7-10 days at 28 °C. *Devosia* sp. grew more slowly on the TYE(A) medium (7-10 days) than the YEM(A) medium (5-7 days). The *Devosia* sp. colonies on the YEM(A) plates appeared a golden yellow color, shiny, mucoid in texture, irregularly shaped, and apparent in non-uniformity of size. However, TYE(A) caused *Devosia* to grow in fairly circular colonies with more uniform size. In addition, *Devosia* colonies on TYE(A) had a smaller and thinner appearance than colonies on YEM(A) (**Figure 1**). A set of results for *Devoisa* growth, based on optical density (OD) measurements on broth culture, is given in **Figure 2**. The results showed that *Devosia* grew faster and more efficiently in YEM(B) medium, under both saline and non-saline conditions than TYE(B) medium (**Figure 2**).

### 3.2 Germination trend

**Figure 3:**
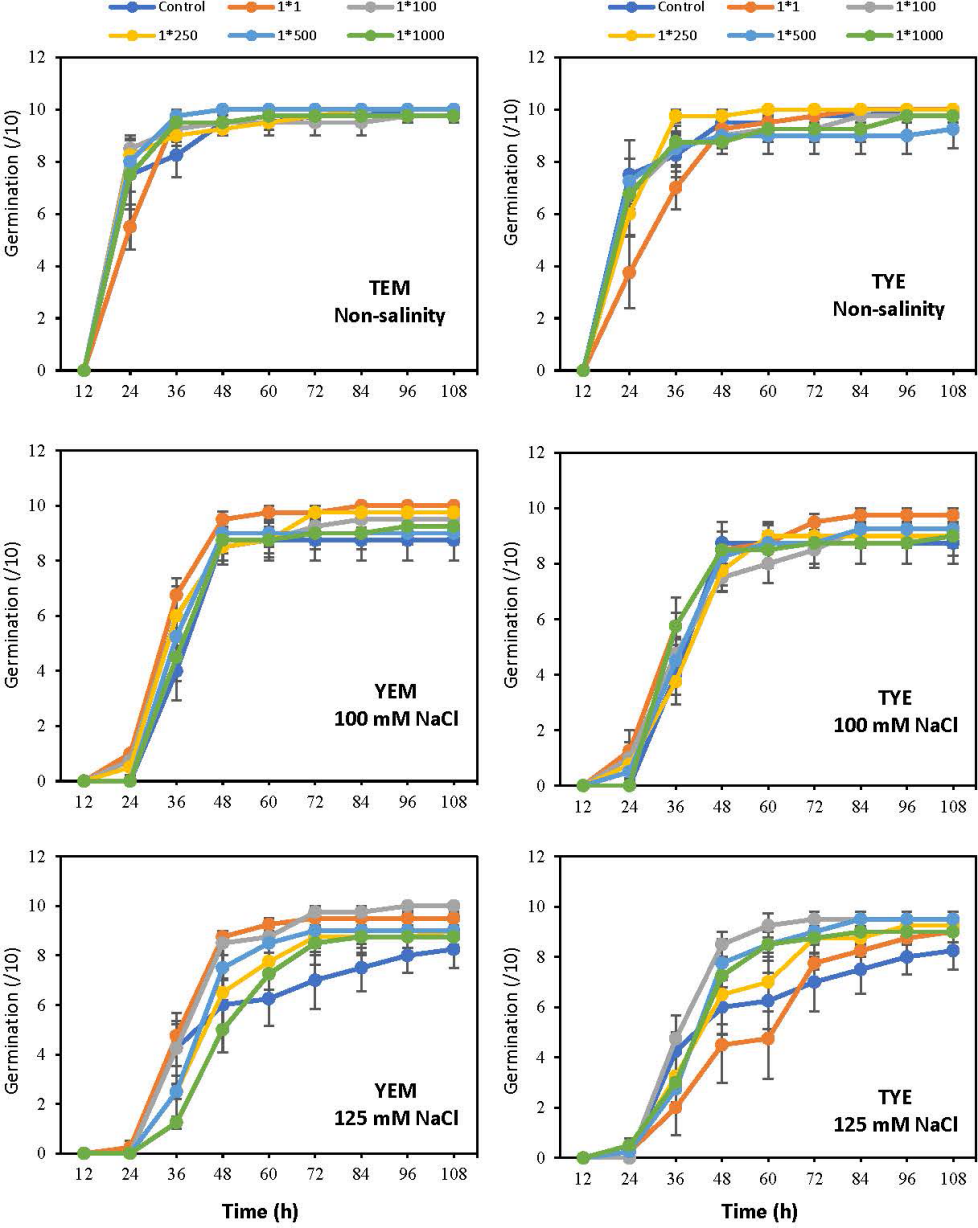
Changes in seed germination for soybean in response to salinity and dilutions of *Devosia* sp. YEM(B)-CFS and TYE(B)-CFS. Values represent the means of four replicates ± SD. 1:1, 1:100, 1:250, 1:500 and 1:1000 = 1 mL *Devosia* sp. CFS and 1, 100, 250, 500 and 100 mL distilled water respectively.

The seed germination trend observed during the study showed that the process was more rapid when YEM(B)-CFS was applied at all salinity levels (**Figure 3**). Using CFS at 1:1 dilution resulted in the quickest germination under 100 mM NaCl. In addition, under the highest salinity level, both 1:1 and 1:100 CFS dilution caused a trend to increased germination than other CFS levels. However, the soybean germination trend was similar under all YEM(B)-CFS levels in non-saline conditions. The application of TYE(B)-CFS showed an increasing trend during soybean germination either in saline or non-saline conditions, and 1:250 (non-saline), 1:1 (100 mM NaCl), and both 1:100 and 1:500 (125 mM NaCl) induced the most rapid seed germination (**Figure 3**).

### 3.3 Final germination

**Figure 4:**
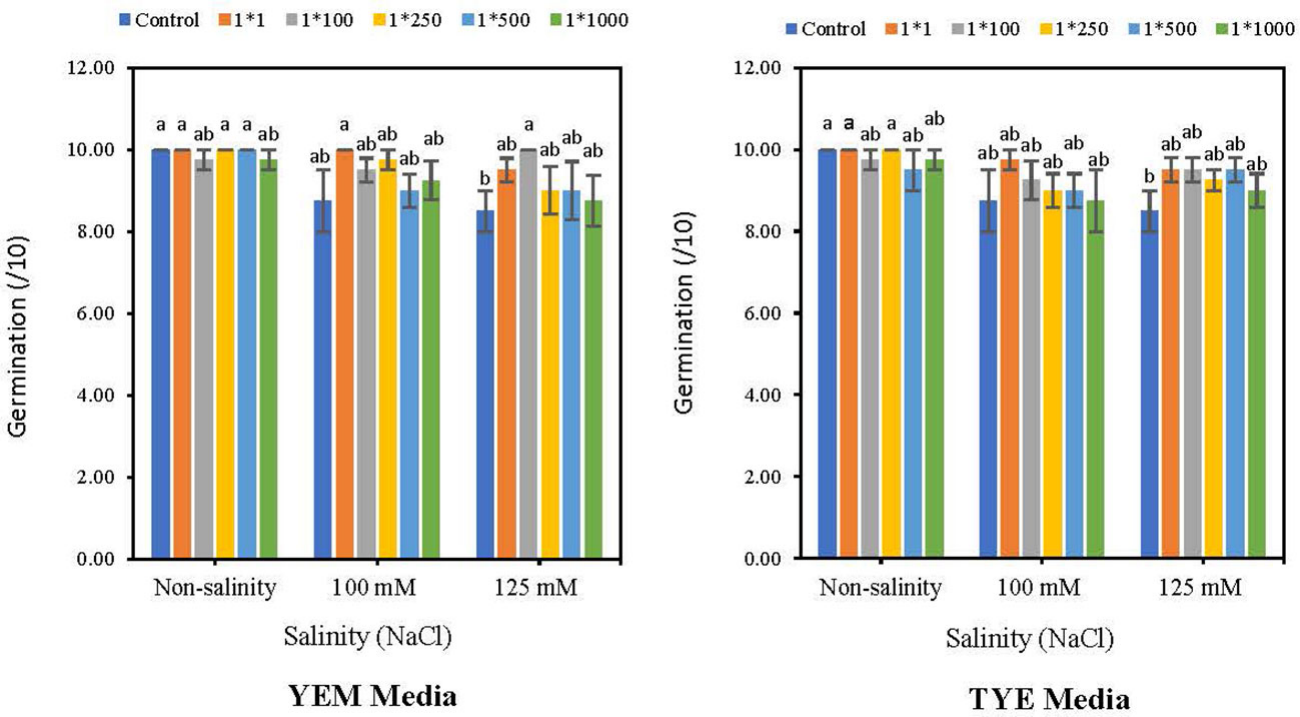
Effect of dilutions of *Devosia* sp. YEM(B)-CFS and TYE(B)-CFS and salinity on germination of soybean seedlings. Values represent the mean of four replicates ± SD. 1:1, 1:100, 1:250, 1:500 and 1:1000 = 1 mL *Devosia* sp. CFS and 1, 100, 250, 500 and 100 mL distilled water, respectively.

The results indicated that *Devosia* YEM(B)-CFS at 1:1 significantly increased soybean germination in the presence of 125 mM NaCl. However, the germination promotion effects of other treatments were not statistically significant (**Figure 4**). In the presence of 125 mM NaCl, YEM(B)-CFS at 1:1 dilution increased the final germination of soybean by 17.6% compared to salt-stressed seeds with no CFS addition. Moreover, under moderate salinity (100 mM NaCl), different levels of YEM(B)-CFS increased final germination from 14.2 (CFS dilution 1:1) to 2.8% (CFS dilution at 1:500);

In contrast, in the presence of 100 mM and 125 mM NaCl, various TYE(B)-CFS levels had no meaningful effect on soybean germination in comparison with salt-stressed seeds. There was only a slight promoting effect from 11.4% (CFS dilution 1:1) to 2.8% (CFS dilution 1:500) under 100 mM NaCl, and from 11.7% (CFS dilutions at 1:1, 1:100, and 1:500) to 5.8% (CFS dilution1:1000) under 125 mM NaCl, compared to stressed seeds with no additional CFS (**Figure 4**).

### 3.4 Germination rate

**Figure 5:**
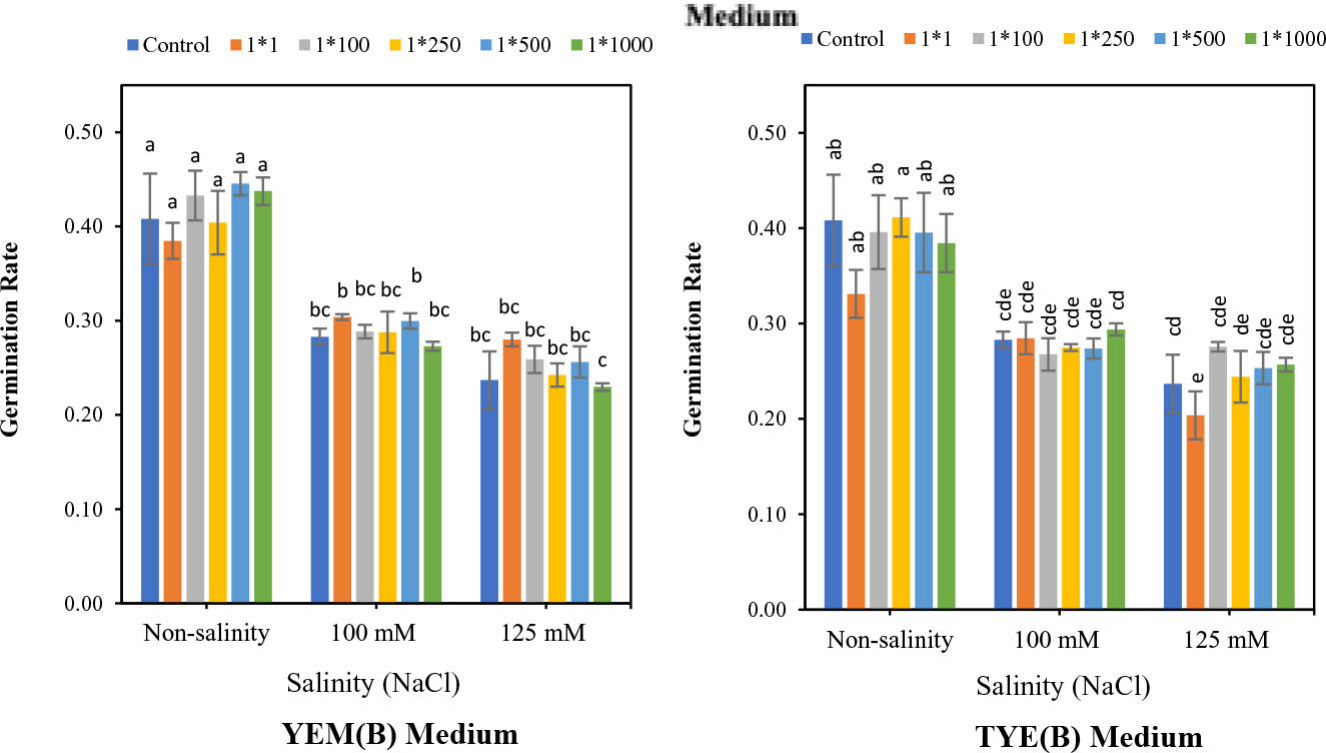
Effect of dilutions of *Devosia* sp. YEM(B)-CFS and TYE(B)-CFS and salinity on germination rate of soybean seedlings. Values represent the mean of four replicates ± SD. 1:1, 1:100, 1:250, 1:500 and 1:1000 = 1 mL *Devosia* sp. CFS and 1, 100, 250, 500, and 100 mL distilled water, respectively.

**Figure 5** indicates that the *Devosia* CFS treatments did not significantly affect the soybean germination rate (GR) of the seeds. However, when seeds were exposed to varying levels of salinity stress, YEM(B)-CFS at 1:1 concentration showed the greatest seed germination enhancement, with a 7.4 (100 mM NaCl) and 18.6% (125 mM NaCl) increase over salt-stressed seeds without CFS application. Moreover, germination enhancement effect of YEM(B)-CFS on GR was limited under non-saline conditions, and YEM(B)-CFS at 1:500 dilutions provided the best GR, with a 9.0% increase over the control seeds.

Application of TYE(B)-CFS had no meaningful effect on soybean GR (**Figure 5**). The maximum enhancement effect of TYE(B)-CFS on GR was only 0.73% at the 1:250 level under the non-salinity conditions, compared to the control seeds. Similarly, GR was not affected by TYE(B)-CFS at 100 mM NaCl. However, at the highest salinity level, the soybean GR was promoted by TYE(B)-CFS from 16.5% (1:100 dilution) to 3.3% (1:250 dilution) compared to the salt-stressed seed with no additional CFS, except for the 1:1 CFS level (**Figure 5**).

### 3.5 Mean germination time

**Figure 6:**
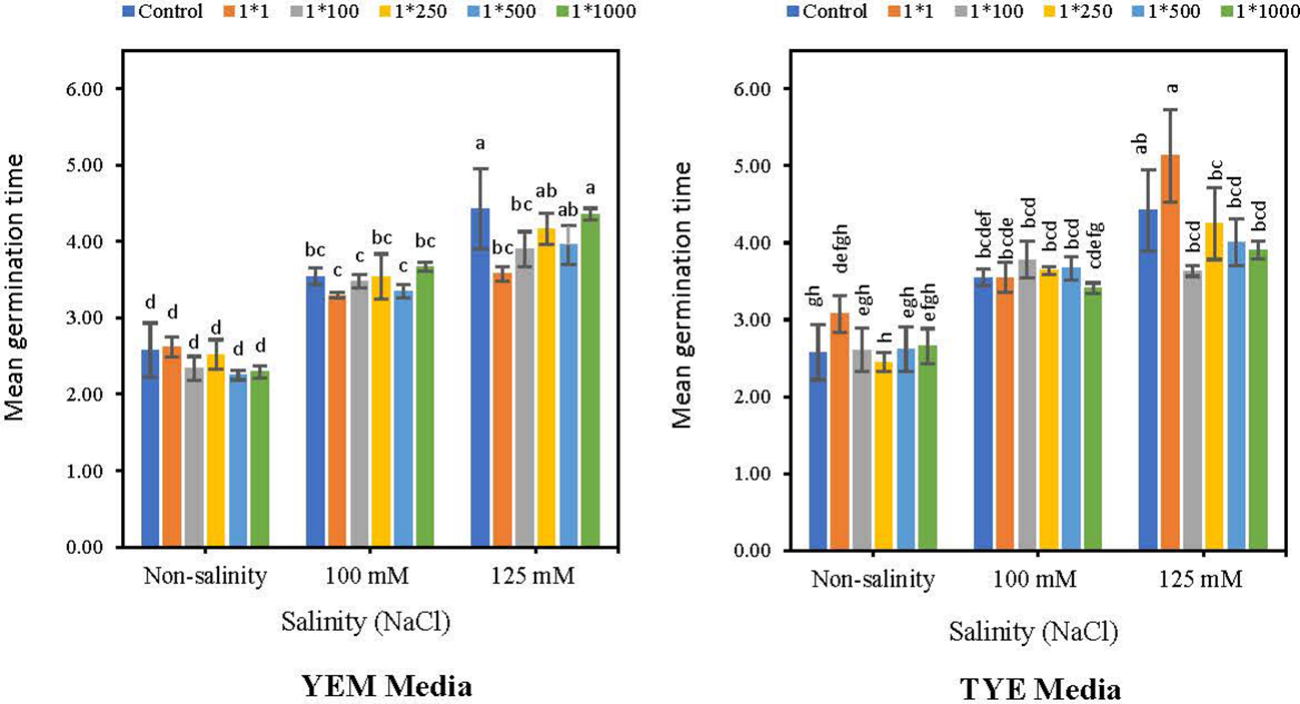
Effect of dilutions of *Devosia* sp. YEM(B)-CFS and TYE(B)-CFS and salinity on mean germination time (MGT) of soybean seedlings. Values represent the mean of four replicates ± SD. 1:1, 1:100, 1:250, 1:500 and 1:1000 = 1 mL *Devosia* sp. CFS and 1, 100, 250, 500 and 100 mL distilled water, respectively.

Applying YEM(B)-CFS caused a meaningful decreased effect on Mean Germination time (MGT) under highest level of salinity (**Figure 6**). The lowest MGT was observed at 1:1 and 1:100 dilutions of YEM-CFS at 125 mM NaCl salinity levels, with 19.2 and 11.7% decreases compared to salt treatments with no addition of CFS. In contrast, under non-stress conditions and 100 mM NaCl salinity level, CFS treatments had no meaningful effect on MGT. Applying YEM(B)-CFS at 1:1 dilution, when seeds were exposed to moderate salinity, only caused a 7% reduction in MGT compared to the stressed seeds with no additional CFS. Additionally, under optimal conditions, YEM(B)-CFS at 1:100 (10.8%), 1:500 (12.4%), and 1:1000 (9.3%) caused a slight reduction in MGT compared to the control seeds.

In addition, applying TYE(B)-CFS under both saline and non-saline conditions had no significant effect on MGT. The highest level of TYE(B)-CFS induced an increase in MGT under non-saline conditions and the highest salinity level, with 19.4 and 16.0% increases, respectively. In addition, at 125 mM NaCl, applying TYE(B)-CFS shortened MGT at 1:100 (17.8%), 1:250 (3.85%), 1:500 (9.5%), and 1:1000 (11.7%) dilutions, compared to the salt-treated seeds without CFS addition (**Figure 6**).

### 3.6 Root length

**Figure 7:**
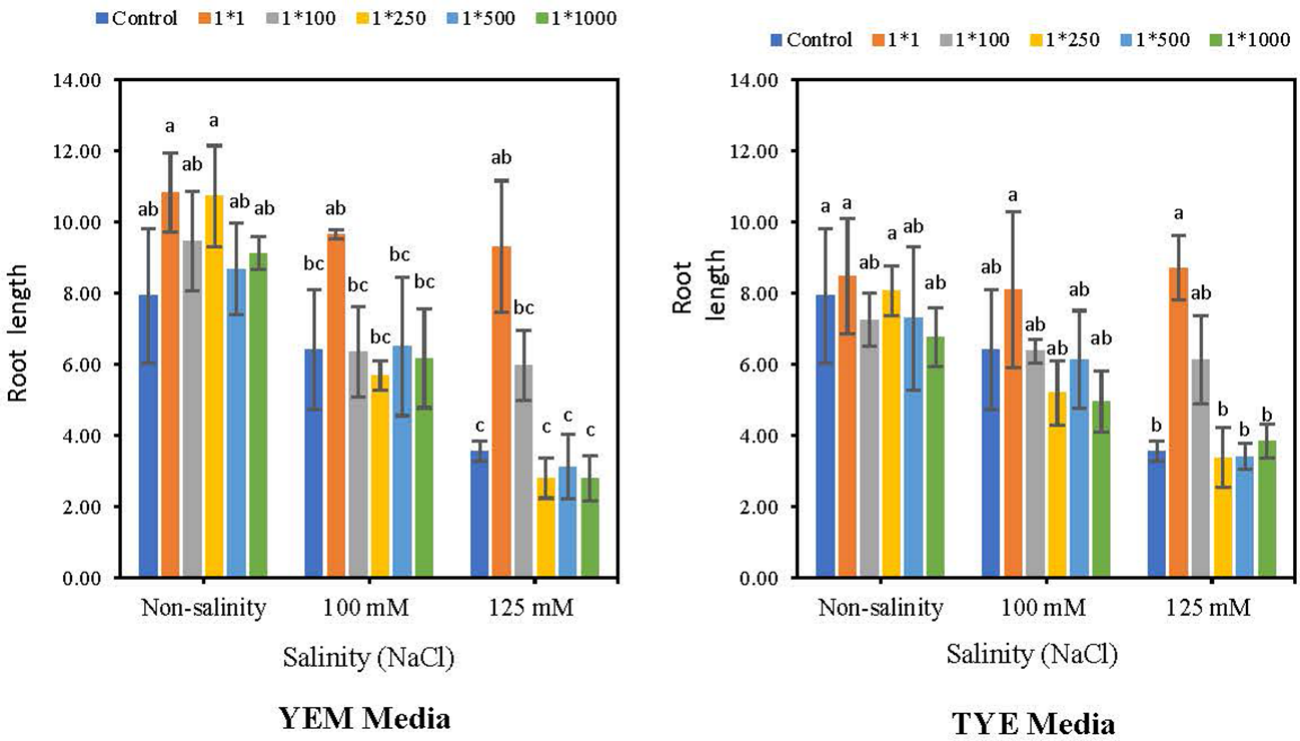
Effect of dilutions of *Devosia* sp. YEM(B)-CFS and TYE(B)-CFS and salinity on root length of soybean. Values represent the mean of four replicates ± SD. 1:1, 1:100, 1:250, 1:500 and 1:1000 = 1 mL *Devosia* sp. CFS and 1, 100, 250, 500 and 100 mL distilled water, respectively.

*Devosia* YEM(B)-CFS and TYE(B)-CFS application significantly increased root length under the highest salinity levels (**Figure 7**). Under 125 mM NaCl condition, applying YEM(B)-CFS at 1:1 and 1:100 dilutions caused the highest increase in soybean root length, by 162.2 and 68.51%, respectively. Under moderate levels of salinity, however, only the 1:1 level of CFS showed the heightening effect with a 50.4% increase compared to the salt-treated seeds with no CFS addition.

Furthermore, applying TYE(B)-CFS at the 1:1 concentration was the best root length promoter with 6.8% (non-saline), 26.0% (100 mM NaCl), and 145.2% (125 mM NaCl) increases; however, only at the highest level of salinity the difference was statistically meaningful. In addition, TYE(B)-CFS at the 1:100 dilution caused an increase in root length under 125 mM NaCl compared to the salt-treated seeds without CFS addition, with 72.6% increase (**Figure 7**).

### 3.7 Root dry weight

**Figure 8:**
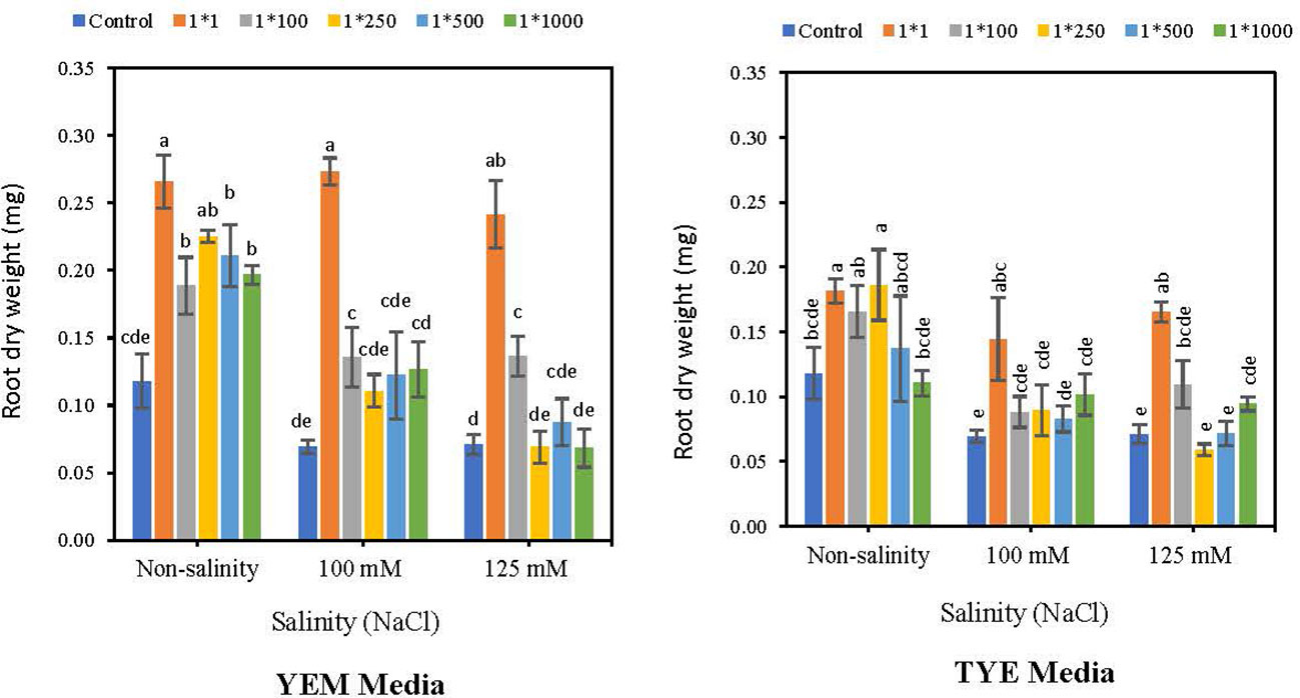
Effect of dilutions of *Devosia* sp. YEM(B)-CFS and TYE(B)-CFS and salinity on Root dry weight of soybean seedlings. Values represent the mean of four replicates ± SD. 1:1, 1:100, 1:250, 1:500 and 1:1000 = 1 mL *Devosia* sp. CFS and 1, 100, 250, 500 and 100 mL distilled water, respectively.

Root weights of soybean seeds were boosted by applying *Devosia*-CFS grown on a YEM(B) culture medium, under both saline and non-saline conditions (**Figure 8**). The results indicated that the highest concentration of YEM(B)-CFS (1:1) had the greatest effect on root weight with 1.26, 2.95, and 2.39 fold increases compared to controls in 0, 100, and125 mM NaCl, respectively. All other concentrations of YEM(B)-CFS showed an elevated effect on root weight, ranging 60.6 to 92.3% under optimal conditions, and 58.9 to 95.0% under 100 mM NaCl. In addition, applying YEM(B)-CFS at 1:100 under extreme salinity levels caused a 91.5% increase in root weight, which was statistically meaningful.

Application of CFS from TYE(B) medium also increased soybean seedling root weight, the 1:1 concentration being the best, with 54.7, 108.6 and 132.3% increases under the 0, 100 mM NaCl, and 125 mM NaCl, respectively. Additionally, TYE(B)-CFS at 1:250 dilution caused a meaningful increase in soybean root dry weight compared to control seeds, by 58.9%. However, the overall results indicated that enhancement effects of *Devosia* CFS from YEM(B) culture medium was greater than from TYE(B) medium (**Figure 8**).

### 3.8 Seed vigor index

**Figure 9:**
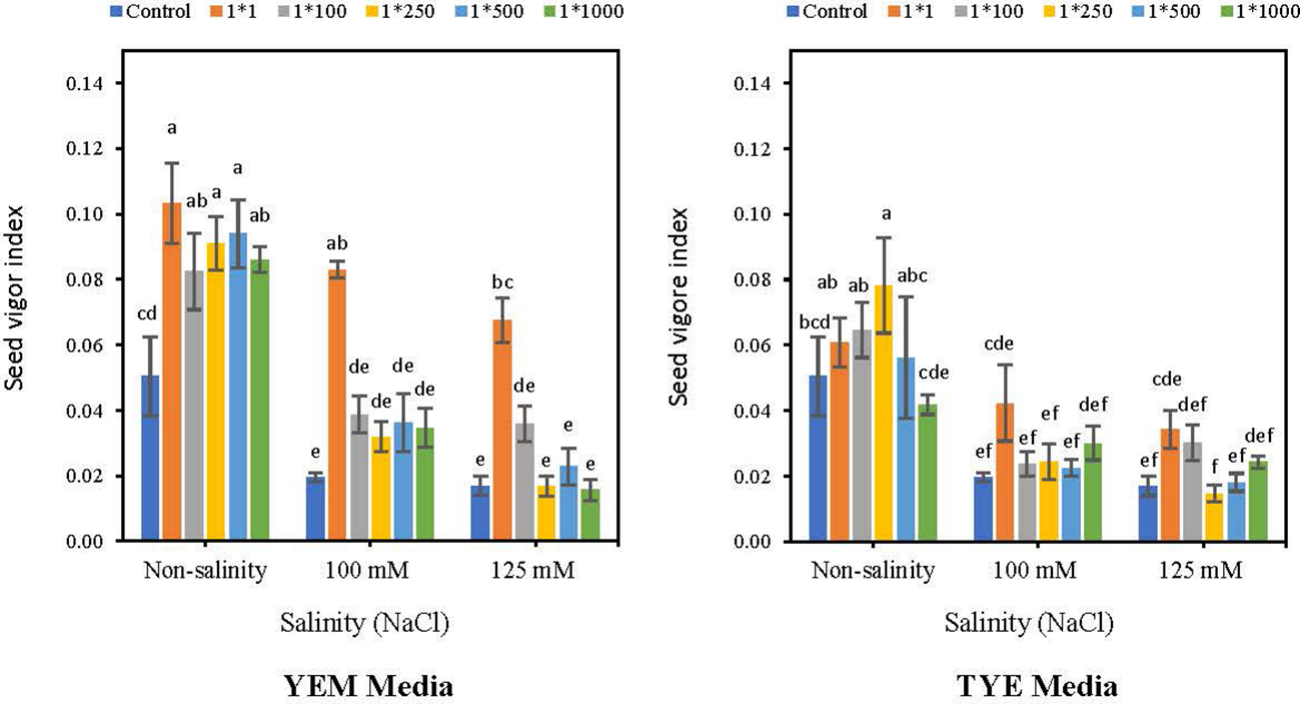
Effect of dilutions of *Devosia* sp. YEM(B)-CFS and TYE(B)-CFS and salinity on seed vigor index of soybean seedlings. Values represent the mean of four replicates ± SD. 1:1, 1:100, 1:250, 1:500 and 1:1000 = 1 mL *Devosia* sp. CFS and 1, 100, 250, 500 and 1000 mL distilled water, respectively.

Applying YEM(B)-CFS caused an increase in seed vigor index (SVI) compared to the control in saline and non-saline conditions (**Figure 9**). However, the boosting effect of CFS was greater when *Devosia* grew in the YEM(B) culture medium. The highest concentration of YEM(B)-CFS (1:1) caused the greatest increases under saline and non-saline conditions with 106% (non-salinity), 336.8% (100 mM NaCl), and 318.7% (125 mM NaCl) enhancements of SVI.

Moreover, the highest SVI resulted from applying a 1:250 concentration of TYE(B)-CFS under non-saline conditions (56%). In addition, TYE(B)-CFS at 1:1 dilution was the best under 100 and 125 mM NaCl which caused a 121.0 and 112.5% increases, respectively; however, these differences were not statistically significant (**Figure 9**).

### 3.9 Correlation

The correlation coefficients of the total number of germinated seeds, GR, MGT, root length, and root dry weight were statistically significant compared with the SVI (**Table 1**). For both YEM(B) and TYE(B) medium, a positive correlation was found between the total number of germinated seeds (r = 0.50 and 0.41, p ≤ 0.01), GR (r = 0.79 and 0.75, p ≤ 0.01), root length (r = 0.84 and 0.71, p ≤ 0.01), and root dry weight (r = 0.92 and 0.90, p ≤ 0.01). However, MGT was negatively correlated with the SVI for both YEM(B) (r = -0.78 p ≤ 0.01) and TYE(B) (r = -0.66, p ≤ 0.01) (**Table 1**).

**Table 1:** The correlation coefficient of laboratory traits and seed vigor index of soybean.

	Seed vigor index	
	YEM(B) Medium	TYE(B) Medium
Final germination	0.5099^**^	0.4135^**^
Mean germination time (MGT)	- 0.7882^**^	-0.6672^**^
Germination rate (GR)	0.7927^**^	0.7554^**^
Root length	0.8432^**^	0.7112^**^
Root dry weight	0.9252^**^	0.9059^**^

ns and **: No significant and significant at p≤0.01, respectively.

## 4 Discussion

PGPB are reliable bioinoculants that improve plant performance, among other things through counteracting salinity stress by biosynthesizing a diverse array of bioactive compounds with the ability to activate and regulate plant physiological mechanisms (Ayuso-Calles et al., 2021). However, many reports suggested that microbial culture medium or nutrition strongly influence bacterial growth and their production of bioactive compounds, and extensive testing of bacterial growth as a function of nutrients is necessary for selecting an appropriate culture medium that can support and promote the growth and survival of microorganisms (Davis et al., 2005; Trianto et al., 2020). For this study, we assessed the effect of a range of growth media for the novel *Devosia* sp. (SL43 strain). Based on the results, we selected yeast-extract-mannitol (YEM) as the culture medium for further experiments, as it resulted in the highest growth rate and cell density either under saline or non-saline conditions, and it was generally more effective for growing *Devosia* SL43 strain than other tested media. In addition, we compared this medium and TYE, which was also effective in producing cultures of the *Devosia* SL43 strain. The results indicated that not only did the bacterium have different growth rates in each medium but also the growth patterns (colony morphology) differed visually. Many reports have indicated that bacterial growth on various media is altered in appearance and maximal growth rates for specific microorganisms, and that specific microorganisms have different growth abilities in the presence of specific nutrients or indicators (Shaneeja et al., 2014; Mohammadkazemi et al., 2015; Wang et al., 2019; Bonnet et al., 2020). Similarly, Xu et al. (2017) reported that colonies of *Devosia nitraria* sp. showed different morphologies and growth abilities in different culture media. In addition, *Devosia* sp. grown on YEM(B) medium, produced more effective results, with regard to soybean germination, than TYE(B) medium. Depending on the bacterial culture medium and nutritional growth requirements of the bacterium, compounds produced by them may exist in different quantities and qualities; it is of interest to facilitate the synthesis of the novel target compounds and validate their activities. Other authors have also demonstrated that differences in the composition of the growth medium affected the biosynthesis of bioactive compounds (Pham et al., 2019; Koim-Puchowska et al., 2021; Yusfi et al., 2021). Plants are generally very sensitive to salinity injury during the germination stage, and exposure to salt stress at early stages can retard seedling growth later, even in conditions otherwise suitable for growth. Soil salinity negatively affects the germination of seeds either by imposing osmotic stress that prevents water uptake or by hormonal imbalance (Kumar et al., 2022; Shah et al., 2022; Yaghoubian et al., 2022a). Our observations indicate that CFS treatment promoted soybean germination under salinity stress conditions (**Figure 4**). The ability of CFS to improve soybean germination under salt stress may result from several mechanisms, such as facilitating resource use or modulating plant hormone levels. Other researchers also reported that the germination of soybean and corn plants under salt conditions was enhanced by application of bacterial cell-free supernatants (Naamala et al., 2022; Shah et al., 2022; Yaghoubian et al., 2022c). Additionally, present results showed that using *Devosia*-CFS accelerated seed germination and stimulated hypocotyl emergence, resulting in a shorter MGT. Promoting effects of CFS may be the result of biologically active substances (hormones, antioxidants, amino acids, vitamins and microbe-to plant signal compounds) produced by the novel *Devosai* SL43 strain, which can improve water up take and regulate germination enzyme activities. It has been reported that inoculation with beneficial microbes provides a broader hormonal pool, which plays a major function in the growth and development of the system (Patten and Glick, 2002; Wang et al., 2012; Tabacchioni et al., 2021). In this sense, lipo-chitooligosaccharides (LCOs) and thuricin 17 are two of the most striking signal compounds investigated so far, both of which were discovered recently; they regulate plant responses to a variety of adverse environmental stresses. In addition, LCOs are produced by N_2_-fixing rhizobacteria following isoflavone induction, triggering formation of nitrogen-fixing nodules in host legumes; thuricin 17 production is constitutive (Lyu et al., 2020). Schwinghamer et al. (2016) reported a positive plant response under saline and temperature stress conditions following from application of thuricin 17 and LCO, causing higher biomass production and root development. It has also been demonstrated that LCO strongly affects the rate and uniformity of canola seed germination under cold conditions, which is critical for early spring seeding under Canadian conditions (Schwinghamer et al., 2015). Correspondingly, Naamala et al. (2022); Yaghoubian et al. (2022b) and Sweeney et al. (2017) reported an enhancing effect of using microbe-derived bioactive compounds in stimulating germination rates of soybean, corn and *Arabidopsis thaliana*, respectively. Another stimulating effect of *Devosia*-CFS was alterations of growth/morphological traits, resulting in heavier and longer roots. Microbial-derived metabolites can influence these traits as a regulator for modulating root traits through increasing acquisition of water and nutrients, either by stimulating lateral root development or affecting osmotic balance. Several studies have demonstrated that many plant-associated microorganisms could profoundly affect root growth (Souleimanov et al., 2002; Sweeney et al., 2017). In addition, Fincheira et al. (2016) demonstrated an increase in dry weight and the number of lateral roots of *Lactuca sativa* independently of the used culture medium. Similarly, Gutiérrez-Luna et al. (2010) suggested that microbe-derived compounds can promote lateral root formation in *Arabidopsis thaliana*. Khan et al. (2011) also showed that lipo-chitooligosaccharides from *Bradyrhizobium japonicum* had a stimulus effect on root growth and development in *Arabidopsis thaliana*. The results clearly indicate that SVI was greatly affected by applying YEM(B)-CFS; SVI is a vital trait for plant establishment and uniformity, and it responded to *Devosia* CFS in a way similar to other measured variables and was positively facilitated by microbe-derived compounds under salinity stress. In general, high soil salinity decreases the SVI, either by creating lower osmotic potentials around the outside of seeds that prevent water uptake or by ionic toxicity stress (Sosa et al., 2005). The application of PGPBs has been proven to be a reliable way of helping plants deal with salinity stress by maintaining the cellular osmotic balance and ion homeostasis. The beneficial effects of PGPB in overcoming osmotic shock after exposure to salt stress can be related to osmolyte accumulation and phytohormone signaling that increases germination uniformity. Our hypothesis regarding the moderating effects of PGPBs on salinity stress is supported by formerly published results (Kang et al., 2014; Egamberdieva et al., 2017; Mishra et al., 2021). Many reports have demonstrated that using PGPB had an increasing effect on germination uniformity and vigor index, helping plants to compete more efficiently under salt stress conditions (Erman et al., 2022; Fan and Smith, 2022). Yaghoubian et al. (2022b) also showed that application of CFS from a *Bacillus* strain caused an increase in the SVI of soybean under salt stress.

## 5 Conclusions

Salinity can be a major abiotic stress in soybean, remarkably reducing the percentage, rate, and uniformity of seedling emergence and, therefore, final soybean production. There is now abundant research suggesting that using plant growth-promoting microbes and their bioactive metabolites is a potentially advantageous approach to improving plant health and productivity under biotic and abiotic stresses. Therefore, gaining detailed knowledge and understanding of cost-effective technologies to produce such biostimulants and biocontrol agents would be important in increasing the availability and/or accessibility of these new products in the agro-input market. This study was focused on choosing an appropriate growth medium for promoting and supporting the growth and survival of the novel *Devosia* sp. strain with the effective bioactive metabolite production, which enhances soybean seed germination under salinity conditions. Findings obtained in this study have suggested that cell-free supernatant obtained from the novel *Devosia* sp. positively improves the germination ability and uniformity of soybean under salt stress. However, increases in seed germination variables were more remarkable when the highest concentration of CFS was applied under salinity stress. Our results also showed that YEM was more appropriate as a growth medium for the *in vitro* cultivation of *Devosai* than TYE medium. The CFS produced by *Devosia* sp. (strain SL43) can successfully reduce salt stress effects on soybean germination variables. It would be interesting to study and manipulate the chemical composition of growth medium to increase bioactive product formation. Another important aspect that would be interesting to be considered is identifying and understanding molecular mechanisms of novel bacterial-derived metabolites which influence host plant physiological and morphological traits and could be a step toward enhancing microbial inoculant technology and developing practical strategies to enhance crop salt tolerance, and quite possibly tolerance to other abiotic stresses.

## Data availability statement

The original contributions presented in the study are included in the article/supplementary material. Further inquiries can be directed to the corresponding author.

## Author contributions

NM conducted the research and did the initial writing. IY assisted in the research and writing. DS provided the intellectual context, the funding and editorial input. All authors contributed to the article and approved the submitted version.
